# Indigo: universal cheminformatics API

**DOI:** 10.1186/1758-2946-3-S1-P4

**Published:** 2011-04-19

**Authors:** D Pavlov, M Rybalkin, B Karulin, M Kozhevnikov, A Savelyev, A Churinov

**Affiliations:** 1 SciTouch LLC, St.-Petersburg, Russia

## 

Indigo is a universal portable open-source library which allows developers and chemists to solve various cheminformatics tasks. The library has grown out from a collection of more specific tools we have developed during the past years. Proper wrappers for popular programming languages are provided, as well as some command-line and GUI tools useful for scientists. All software is available under the terms of the GPL license.

The core of Indigo is a C++ cheminformatics library, which has been developed with two things in mind: performance and important chemical features. Plain C wrapper is provided for the core library, and other wrappers are built around it for Python, Java, and C# languages (support of Ruby is coming soon). The main chemical features of Indigo are:

• Support of popular chemistry formats: Molfiles/Rxnfiles v2000 and v3000, SDF, RDF, SMILES, SMARTS, SMIRKS

• Tehrahedral and cis-trans stereochemistry support

• Molecule and reaction rendering to PNG, SVG, PDF files

• Molecule and reaction layout (depiction)

• Aromatization and kekulization

• Canonical (isomeric) SMILES computation

• Exact and substructure matching for molecule and reactions, SMARTS and SMIRKS

• matching, highlighting support

• Matching or tautomers and resonance structures

• Molecule and reaction fingerprinting, similarity computation

• Molecular weight, molecular formula computation

• Maximum Common Substructure (MCS) computation

• R-Group deconvolution and scaffold detection

• Combinatorial chemistry

The pre-compiled static and dynamic binaries of the Indigo library are available, as well as binaries of command-line utilities and GUI tools. The library allows multi-threaded use. All binaries are provided for Windows, Linux and Mac OS X, both 32-bit and 64-bit Intel architectures.

The site of Indigo is http://scitouch.net/indigo (to be opened in August 2010).

